# *Bletilla striata* (Orchidaceae) Seed Coat Restricts the Invasion of Fungal Hyphae at the Initial Stage of Fungal Colonization

**DOI:** 10.3390/plants8080280

**Published:** 2019-08-11

**Authors:** Chihiro Miura, Miharu Saisho, Takahiro Yagame, Masahide Yamato, Hironori Kaminaka

**Affiliations:** 1 Faculty of Agriculture, Tottori University, 4-101 Koyama Minami, Tottori 680-8553, Japan; 2 Mizuho Kyo-do Museum, 316-5 Komagatafujiyama, Mizuho, Tokyo 190-1202, Japan; 3 Faculty of Education, Chiba University, 1-33 Yayoicho, Inage-ku, Chiba 263-8522, Japan

**Keywords:** *Bletilla striata*, mycorrhizal symbiosis, Orchidaceae, seed coat, seed morphology, symbiotic germination

## Abstract

Orchids produce minute seeds that contain limited or no endosperm, and they must form an association with symbiotic fungi to obtain nutrients during germination and subsequent seedling growth under natural conditions. Orchids need to select an appropriate fungus among diverse soil fungi at the germination stage. However, there is limited understanding of the process by which orchids recruit fungal associates and initiate the symbiotic interaction. This study aimed to better understand this process by focusing on the seed coat, the first point of fungal attachment. *Bletilla striata* seeds, some with the seed coat removed, were prepared and sown with symbiotic fungi or with pathogenic fungi. The seed coat-stripped seeds inoculated with the symbiotic fungi showed a lower germination rate than the intact seeds, and proliferated fungal hyphae were observed inside and around the stripped seeds. Inoculation with the pathogenic fungi increased the infection rate in the seed coat-stripped seeds. The pathogenic fungal hyphae were arrested at the suspensor side of the intact seeds, whereas the seed coat-stripped seeds were subjected to severe infestation. These results suggest that the seed coat restricts the invasion of fungal hyphae and protects the embryo against the attack of non-symbiotic fungi.

## 1. Introduction

Orchidaceae is one of the largest and most diverse families of flowering plants, comprising approximately 25,000 species [1,2,3]. One of the family’s most important characteristics is that the orchids in it produce minute seeds that contain limited or no endosperm, and, therefore, they must form an association with symbiotic fungi to obtain nutrients during germination and subsequent seedling (protocorm) growth under natural conditions [4]. The minute seed consists of an ovoid or spherical embryo surrounded by a thin seed coat. The seed coat is generally fusiform and (semi-)transparent, with variable surface patterns depending on the genus or species [5]. The various ultrastructures of orchid seeds are frequently considered to be taxonomically useful at the higher taxonomic level [1,6]. These structures give rise to an increase in air resistance, allowing seeds to remain air-borne or to float on water for long periods [7], although we still have limited understanding of the functional and ecological consequences of this structural variation [7,8].

In nature, orchid seed germination requires compatible symbiotic fungi that are free-living saprotrophs or ectomycorrhizal fungi belonging to Basidiomycota or Ascomycota [9]. Symbiotic fungal hyphae penetrate orchid seeds either through the suspensor [10,11,12] or through epidermal hairs [13] and then form dense coils of mycelium called pelotons. The symbiotic protocorms limit the fungal growth to cortical cells at the suspensor side of the embryo, and the pelotons are not found in the apical meristem [14,15]. It is generally accepted that orchid cells obtain nutrients including carbon compounds thorough the symbiotic cells, predominantly during peloton degradation [16].

In contrast to mutual and collaborative symbiotic relationships, such as arbuscular mycorrhizal symbiosis, orchid mycorrhizal relationships are generally unstable. Symbiotic fungi can potentially become pathogenic parasites or fail to colonize the protocorms when the temperature or nutritional composition is not suitable for symbiosis [17,18]. The orchid mycorrhizal fungi have the potential to secrete a variety of cell wall-degrading enzymes that induce plant immunity [19]. Association with symbiotic fungi carries a risk of causing serious damage to orchids [17,20,21]. Shimura et al. [22] reported that the orchid *Cypripedium macranthos* var. *rebunense* produced two antifungal compounds, suggesting that orchid plants equip themselves with these compounds to protect them against the fungal invasion. Moreover, natural soils contain a vast diversity of microorganisms, including both symbiotic and pathogenic fungi [23]. It is a prerequisite for orchids to select an appropriate fungus among the diverse soil fungi and establish a suitable symbiosis at the germination stage. Thus, recruiting appropriate orchid mycorrhizal fungi as symbiotic partners is indispensable to initiate the symbiotic seed germination.

In this study, we aimed to better understand the process of recruitment of symbiotic fungi at the symbiosis initiation stage, focusing on the orchid seed coat since the seed coat is the first point of fungal attachment. To test this, we used an experimental system for a symbiotic germination of a terrestrial orchid, *Bletilla striata* (subfamily Epidendroideae, tribe Arethuseae), on an oatmeal medium [24]. The seed material was chosen because it has a relatively large air space, leading to easy handling. *B. striata*, is usually found on sunshiny moist grassland and known as a common garden plant in Japan, which produces numerous dust-like seeds (approximately 0.2 mm-long embryo and 1.0 mm-long seed coat). When the symbiotic germination occurs, the embryo swells, epidermal hairs develop within a week, and then the shoot apical meristem of most seeds emerges within two weeks, after the seed coat has ruptured [24]. Since *B. striata* cells containing pelotons are present in one-week-old protocorms [24], the fungal hyphae are assumed to reach the surface of the embryo through the seed coat in a few days. Consequently, we examined the role of the seed coat in the initial stage of the interaction with symbiotic and pathogenic fungi by comparing intact *B. striata* seeds with seeds from which the seed coat had been removed under a stereomicroscope. Our results reveal that both symbiotic and pathogenic fungi heavily invade the seed coat-stripped seeds, suggesting that the seed coat protects the embryo against fungal attack.

## 2. Results

### 2.1. Effect on Germination of Inoculating Bletilla Striata Seeds Stripped of Seed Coat with the Symbiotic Fungus Tulasnella sp. Strain HR1-1

**Figure 1:**
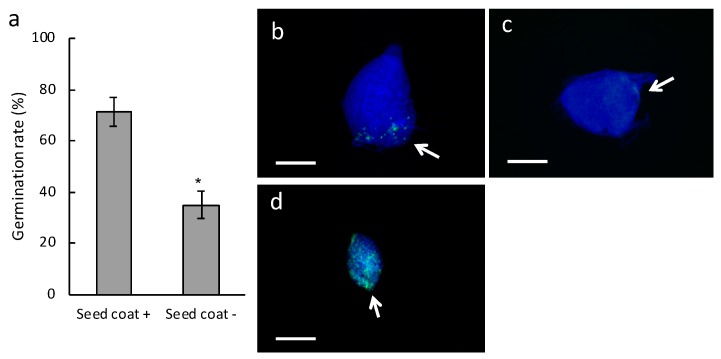
Seed germination of *Bletilla striata* inoculated with the symbiotic fungus *Tulasnella* sp. strain HR1-1. (**a**) Germination rate of *B. striata* seeds. Gray bars indicate the germination rate defined as the emergence of shoot apex at two weeks after sowing. Seed coat +, intact seeds; Seed coat −, seed coat-stripped seeds. Error bars represent standard errors of the means of five independent experiments. The asterisk shows significant difference between the treatments as determined by the Student’s *t*-test at *p* < 0.01. (**b**–**d**) Fungal colonization of *B. striata* seeds by *Tulasnella* sp. strain HR1-1. The images show two-week-old protocorms that were stained with calcofluor white (blue) and wheat germ agglutinin (WGA)-Alexa fluor-488 (green) to visualize the plant cell and fungal structures, respectively. (**b**) A successfully germinated intact seed. (**c**) An ungerminated intact seed. (**d**) A seed coat-stripped seed. Arrows indicate the suspensor end. Scale bars, 200 µm.

To understand the role of the seed coat of *B. striata*, we compared the germination rate between intact seeds and seed coat-stripped seeds when they were inoculated with the symbiotic fungus *Tulasnella* sp. strain HR1-1. The germination rate was significantly lower for the stripped seeds than for the intact seeds two weeks after sowing (Figure 1a). When the stripped seeds were sown on Hyponex–sucrose agar medium for asymbiotic germination, they grew as well as (or slightly better than) intact seeds (Figure S1), indicating that the seed embryos were viable and non-damaged after the stripping. These results reveal that the seed coat plays a key role in symbiotic interactions.

Fluorescence microscopy showed that intact seeds which successfully germinated contained green fluorescent dots at the suspensor side of the embryo, indicating the presence of fungal pelotons (Figure 1b), but fungal hyphae were arrested at the suspensor end in intact seeds that did not germinate (Figure 1c). On the other hand, proliferated fungal hyphae were observed inside and around ungerminated seed coat-stripped seeds, and no peloton structures was found (Figure 1d).

### 2.2. Germination Rate and Fine Observations of Seed Coat-Stripped Seeds of Bletilla striata Inoculated with the Symbiotic Fungus Sebacina Vermifera

**Figure 2:**
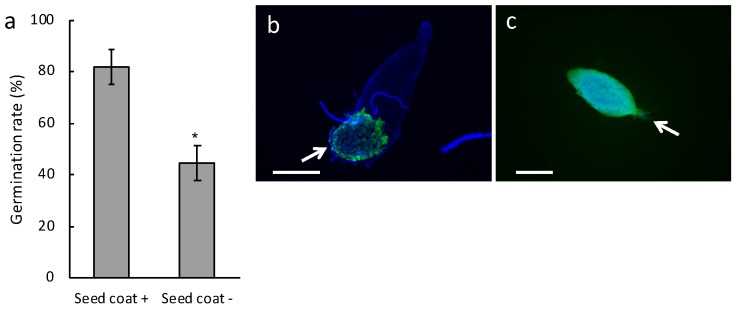
Seed germination of *Bletilla striata* inoculated with the symbiotic fungus *Sebacina vermifera*. (**a**) Germination rate of *B. striata* seeds. Gray bars indicate the germination rate defined as the emergence of shoot apex at two weeks after sowing. Seed coat +, intact seeds; Seed coat −, seed coat-stripped seeds. Error bars represent standard errors of the means of five independent experiments. The asterisk shows significant difference between the treatments as determined by the Student’s *t*-test at *p* < 0.01. (**b**,**c**) Colonization of *B. striata* seeds by *S. vermifera*. The images show two-week-old protocorms that were stained with calcofluor white (blue) and WGA-Alexa fluor-488 (green) to visualize the plant cell and fungal structures, respectively. (**b**) A successfully germinated intact seed. (**c**) A seed coat-stripped seed. Arrows indicate the suspensor end. Scale bars, 500 µm (**b**) and 200 µm (**c**).

To confirm the role of the *B. striata* seed coat in symbiotic interactions, we performed similar analyses using another symbiotic fungus, *S. vermifera* strain 0723. Although *S. vermifera* is not a natural symbiont of *B. striata*, fungal taxa of the genus Sebacina are described as generalists and ubiquitously distributed [25]. *B. striata* seeds sown in association with *S. vermifera* successfully germinated on an oatmeal agar medium and formed pelotons within symbiotic cells (Figure S2a,b). This confirmed that *S. vermifera* acted as a symbiotic partner of *B. striata*. A comparison analysis of *B. striata* seeds with or without seed coats revealed that the germination rate of seed coat-stripped seeds inoculated with *S. vermifera* was significantly lower than for intact (Figure 2a), reflecting the result with *Tulasnella* sp. strain HR1-1.

**Figure 3:**
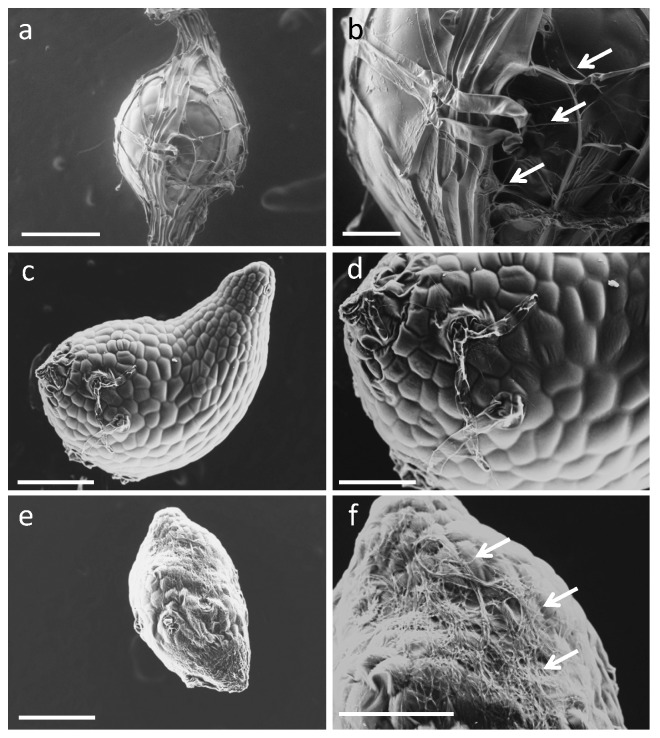
Scanning electron microscopy (SEM) images of one-week-old *Bletilla striata* protocorms inoculated with the symbiotic fungus *Sebacina vermifera*. (**a**,**b**) Intact seed with seed coat retained, (**c**,**d**) intact seed with peeled off just before observation, and (**e**,**f**) a seed coat-stripped seed. Arrows indicate fungal hyphae. Scale bars, 200 µm (**a**,**c**,**e**), 50 µm (**b**), and 100 µm (**d**,**f**).

Fluorescent microscopy showed that the successfully germinated seeds in intact seeds apparently contained peloton structures (Figure 2b), while, in contrast, invasive hyphae with no peloton structures were found both at the suspensor end and the central part of the embryo in ungerminated seed coat-stripped seeds (Figure 2c). Successfully germinated seed coat-stripped seeds at low frequencies contained peloton structures (Figure S2c). To further visualize the initial fungal behavior, we conducted a scanning electron microscopy (SEM) study of one-week-old protocorms. Although fungal hyphae were observed on the surface of the seed coat of symbiotically germinated seeds (Figure 3a,b), no visible hyphae were found around the embryos when the seed coat was removed from the intact seed just before the SEM observation (Figure 3c,d). Conversely, the seeds which were exposed to *S. vermifera* after stripping of the seed coat showed numerous hyphae on the surface of the embryos (Figure 3e,f). These results indicate that the seed coat restricted the spread of fungal hyphae on the embryo.

### 2.3. Infection Rate of Seed Coat-Stripped Seeds of Bletilla striata Inoculated with Pathogenic Fungi Rhizoctonia Solani and Fusarium Oxysporum

**Figure 4:**
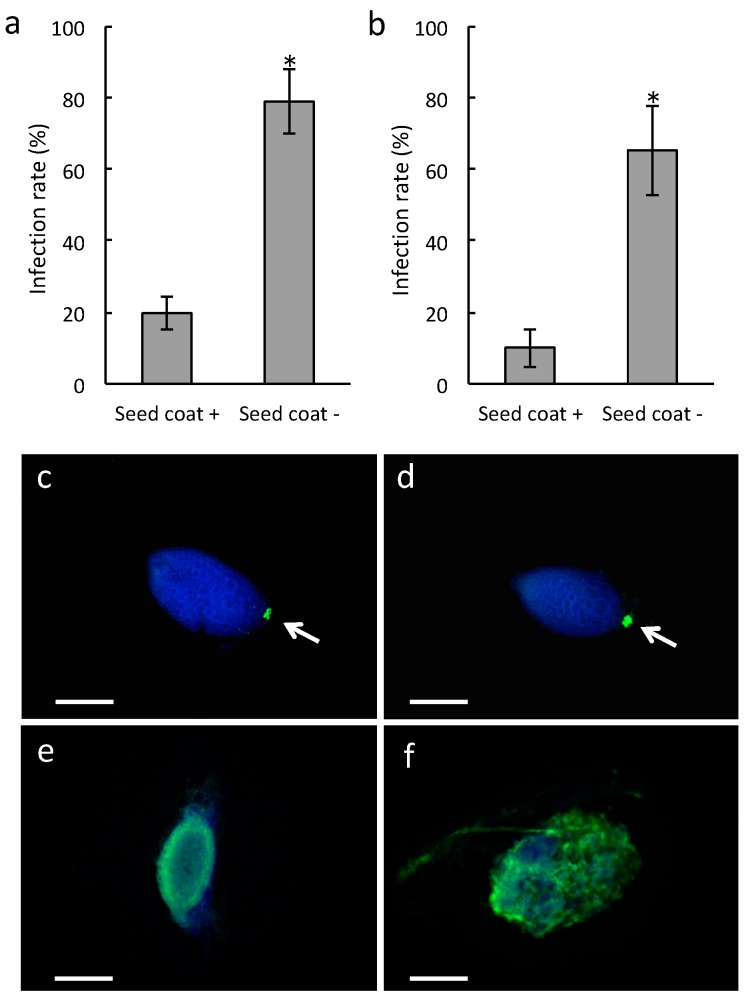
*Bletilla striata* seeds inoculated with pathogenic fungi. *B. striata* seeds were inoculated with *Rhizoctonia Solani* (**a**,**c**,**e**) or *Fusarium oxysporum* (**b**,**d**,**f**). (**a**,**b**) Infection rate of *B. striata* seeds. Gray bars indicate the infection rate defined as hyphal proliferation at three weeks after sowing (see also Figure S3). Seed coat +, intact seeds; Seed coat −, seed coat-stripped seeds. Error bars represent standard errors of the means of four independent experiments. The asterisk shows significant difference between the treatments as determined by the Student’s *t*-test at *p* < 0.01. (**c**–**f**) Colonization of *B. striata* seeds by *R. solani* or *F. oxysporum*. The images show three-week-old protocorms that were stained with calcofluor white (blue) and WGA-Alexa fluor-488 (green) to visualize the plant cell and fungal structures, respectively. (**c**,**d**) Intact seeds; (**e**,**f**) seed coat-stripped seeds. Arrows indicate the suspensor end. Scale bars, 200 µm.

Our findings lead to the hypothesis that the seed coat could play a role in guiding fungal invaders to *B. striata* cells at the suspensor end to prevent pathogenic fungi from causing infections. To determine if this was true, we exposed intact and seed coat-stripped seeds to two causal agents of various plant diseases, *Rhizoctonia Solani* Kuhn AG-3 and an incompatible pathogen *Fusarium oxysporum* f. sp. Lycopersici, and compared the infection rates. Co-culture of *B. striata* seeds that lacked their seed coat with either *R. solani* or *F. oxysporum* resulted in an increase in the infection rate (Figure 4a,b). Fungal hyphae were detected only in the suspensor side of intact seeds inoculated with either *R. solani* or *F. oxysporum*, (Figure 4c,d), whereas the seed coat-stripped seeds experienced severe infestation by these fungi (Figure 4e,f). These results support our hypothesis that the seed coat could protect the embryo against fungal attack.

### 2.4. Fine Observation of the Bletilla striata Seeds Inoculated with Symbiotic Fungi

**Figure 5:**
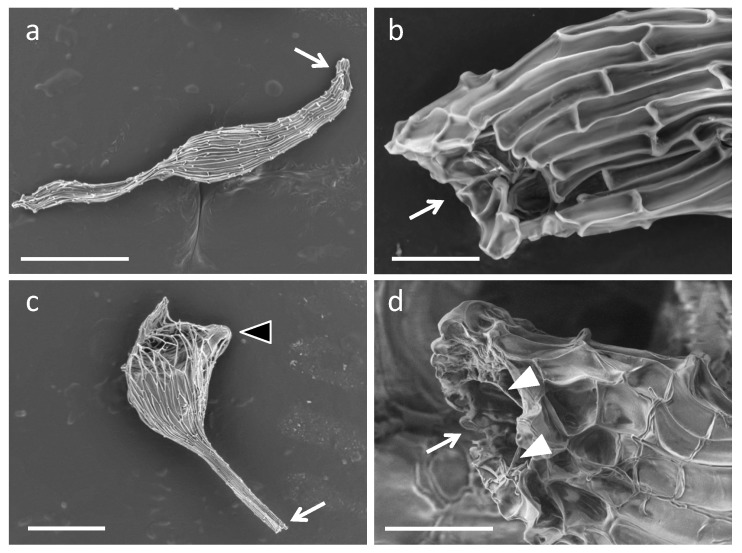
SEM images of *Bletilla striata* inoculated with a symbiotic fungus *Tulasnella* sp. strain HR1-1. The images show *B. striata* seeds before sowing (**a**,**b**) and the germinated seeds inoculated with *Tulasnella* sp. strain HR1-1 (**c**,**d**). Arrows indicate a hole at the end of seed coat. The black arrow head indicates a shoot apical meristem. The white arrow heads indicate fungal hyphae. Scale bars, 500 µm (**a**,**c**) and 50 µm (**b**,**d**).

Since the symbiotic fungal hyphae directly surrounded the seed coats (Figure 3a,b), it was presumed that the seed coats were not exhibiting antifungal activity. Accordingly, we postulated that the seed coat structure may have been contributing to resistance against fungal penetration. SEM revealed the existence of a hole at the one end of the seed coat of *B. striata* seeds (Figure 5a,b), and the hole existed at the side of the suspensor end (Figure 5c). Furthermore, fungal hyphae existed at the hole entrance when *B. striata* seeds were grown with *Tulasnella* sp. strain HR1-1 (Figure 5d).

## 3. Discussion

Hyphae of compatible fungi penetrate orchid seeds either through the suspensor [10,11,12] or through epidermal hairs [13] and then form pelotons. The present study showed that seed coat-stripping treatment of *B. striata* seeds resulted in a low germination rate and pathogen-like infection, even following inoculation with the symbiotic fungi *Tulasnella* sp. strain HR1-1 and *S. vermifera* (Figure 1 and Figure 2). Moreover, when intact seeds were inoculated with the pathogenic fungi *R. solani* and *F. oxysporum*, hyphae were restricted to the suspensor end. These results suggest that the *B. striata* seed coat plays a role in restricting ingress of fungal hyphae into the embryo cells.

Chemical and physical defense in plant seeds are likely to be shaped by interactions with seed pathogens, which have a major impact on seed survival in the soil [26]. In terrestrial orchids, the outer seed coat enclosing the embryo has been reported to contain lignin, suberin, polyphenols, lipids, and cuticular materials [8,27,28,29], implying a hydrophobic nature. Some physical mechanisms of the seed coat, such as restriction of water uptake, could be one of the reasons behind germination inhibition since sonication treatment has been shown to enhance seed germination of *Calanthe discolor* [30]. The present study is congruent with this explanation; the germination of seed coat-stripped seeds of *B. striata* was slightly (but not significantly) increased in the case of asymbiotic germination (Figure S1). Seed-enclosing structures are also generally considered to be sufficiently restrictive to microbial access to the seed embryo [26]. Taking into account our finding that the fungal hyphae did not directly surround the *B. striata* embryo but did surround the seed coat, it appears that the seed coat protects the seeds from fungal infection physically rather than chemically.

The seed morphologies of various kinds of orchids have been studied, with the aim of more precise classification of the family [5,31,32,33]. These studies have revealed remarkable commonalities and yet also the diversity of seed morphology. Although knowledge of its functional role is limited, it is generally believed that morphological varieties of orchid seed coats relate to the dispersal strategy. For example, the surface sculpting could increase air resistance; almost all orchid seeds share common features of adaptation to wind dispersal [7]. High hydrophobicity confers seed flotation in the genus *Disa*, enabling seed dispersal along streams [34]. The highly lignified seed coat in the genera *Vanilla* and *Cyrtosia* [35,36] is considered to be an adaptation to animal dispersal [36,37]. Previous morphological studies have also shown that the orchid seed coat forms a sac-like structure [4,5,34,38] and have suggested that the opening of the seed coat is wide enough to allow entry of water and hyphae [4]. Our results support this suggestion (Figure 5) and imply that the fungal hyphae reach the suspensor end through the hole in the seed coat, although further investigations are needed to determine the primary route of fungal entry.

It is generally accepted that orchid mycorrhizal relationships are unstable; symbiotic fungi could potentially become pathogenic or fail to colonize the protocorms when the temperature or carbon source are not suitable for symbiosis [15,17,18]. The present study showed that pathogen-like infection occurred in parts of ungerminated seed coat-stripped seeds following inoculation with symbiotic fungi (Figure 1 and Figure 2). This result is reminiscent of the inherent nature of orchid mycorrhizal fungi that secrete a variety of cell wall-degrading enzymes typically associated with saprotrophism [19]. In contrast, successfully germinated coatless seeds at low frequencies inoculated with *S. vermifera* contained peloton structures. There could be a certain probability of successful germination occurring in the coatless seeds because fungi can attach on the coatless seeds from any direction, which, of course, includes the suspensor end. The symbiotic germination process could begin when the fungi attack the suspensor end initially, although it is unclear why the coatless seeds are not harmed anymore. Since the germination of *B. striata* seeds appears to require quite a delicate balance between fungal symbiosis and pathogenesis, a well-defined mechanism for reaching the suspensor end and/or the restriction of the entry point to the suspensor end by seed coats is important to promote the establishment of the symbiotic relationship.

Orchids are an evolutionarily successful plant group with extraordinary species diversity. Although this success indicates that the potential benefits of producing many dust-like seeds, which consist only of an undifferentiated embryo and an apparently rather brittle seed coat, outweigh the costs of that, such minute seeds also harbor great potential for their survival, for example, complex requirements for germination, seed longevity, and effective seed dispersal mechanisms [37,39,40,41]. In this study in *B. striata*, we have uncovered a new and different functional role for the seed coat in establishing the symbiotic relationship, which provides a barrier for plant tissue subjected to various types of fungal infection. A similar analysis of other forms of orchid seed would help improve understanding of the selection mechanism of the right partner among the soil microbiota and the potential adaptive significance of species diversity in orchids.

## 4. Materials and Methods

### 4.1. Plant Materials and Fungal Strains

Seeds of *B. striata* ‘Murasakishikibu’ used as the plant material in this study were prepared as described previously [24]. Collected seeds were stored at 4 °C until use. Two fungal species, *Tulasnella* sp. strain HR1-1 [24] and *S. vermifera* strain 0723 (MAFF305830), obtained from the Genetic Resources Center, National Agriculture and Food Research Organization, Japan were used for symbiotic germination as symbiotic fungi of *B. striata*, while two other fungi, *R. solani* Kuhn AG-3 (ATCC MYA-4579), obtained from ATCC (https://www.atcc.org/en.aspx), and *F. oxysporum* f. sp. *lycopersici* strain JCM 12575, provided by Dr. T. Arie, were used for infection assays as the pathogenic fungi. Fungal strains of *Tulasnella* sp., *R. solani*, and *F. oxysporum* were maintained on potato dextrose agar (BD Difco, NJ, USA), and *S. vermifera* was maintained on 1/6-strength Czapek Dox agar medium containing 0.83 g/L yeast extract [42].

### 4.2. Seed Inoculation

Seed inoculation with symbiotic or pathogenic fungi was performed according to the method of Yamamoto et al. [24], with slight modifications as follows. Fungal strains were precultured on oatmeal agar (OMA) medium (2.5 g/L OMA (BD Difco), 6.5 g/L agar) for one week at 25 °C in the dark prior to seeding. Surface-sterilized *B. striata* seeds were placed on 0.1% sterilized water–agar medium, and to expose the embryo, the seed coat was removed from each seed using a dissecting needle under a stereomicroscope (SW-700TD; Wraymer, Osaka, Japan) in a clean booth. The seed coat-stripped seeds were sown onto precultured fungal hyphae on OMA side-by-side with intact seeds. *B. striata* seeds were co-cultured with symbiotic fungi *Tulasnella* sp. and *S. vermifera* for two weeks at 25 °C in the dark, and with pathogenic fungi *R. solani* and *F. oxysporum* for three weeks at 25 °C in the dark. For asymbiotic germination, the surface-sterilized seeds were placed on Hyponex–agar medium (3.0 g/L Hyponex powder (Hyponex Japan, Osaka, Japan), 2.0 g/L peptone, 30 g/L sucrose, 10 g/L agar) for three weeks at 25 °C in the dark.

### 4.3. Evaluation of Seed Germination and Fungal Infection

The seed germination rate of *B. striata* seeds inoculated with *Tulasnella* sp. and *S. vermifera* was defined as the emergence of a shoot according to Yamamoto et al. [24]. *R. solani* and *F. oxysporum* infections were determined by visible symptoms (Figure S3). In *R. solani*-infected seeds, the fungal hyphae covered the surface of the seed, and the seed color was creamy white or brown. In *F. oxysporum*-infected seeds, the fungal hyphae covered the surface of the seed. The seed color changed from creamy white to dark purple or brown. At least 13 pairs of intact and seed coat-stripped seeds were studied in one series of experiments. These experiments were independently repeated three–four times.

### 4.4. Fluorescent Staining of Fungal Hyphae

The staining procedure was performed as previously described [24], with slight modifications, as follows. After neutralization of potassium hydroxide (KOH) treatment, the seeds were stained with 40 µg/mL WGA-Alexa fluor-488 (Alexa-488) solution (Thermo Fisher Scientific, Waltham, MA, USA) for 15 min and 20 µg/mL Calcofluor White solution (Sigma-Aldrich, St. Louis, MO, USA) for 5 min in the dark at room temperature. A seed coat of the stained intact seed was removed under a stereomicroscope (SZX16; Olympus, Tokyo, Japan). Stained cells were observed under a fluorescence microscope (DM2500; Leica, Wetzlar, Germany) with an excitation filter of 480/40 nm for Alexa-488-conjugated fungal hyphae and 340–380 nm for Calcofluor White-stained plant cell walls.

### 4.5. Scanning Electron Microscope Observations

*B. striata* seeds were mounted on a carbon tape and observed using a low vacuum SEM (TM3030Plus; Hitachi High-Technologies Corporation, Tokyo, Japan). The intact seeds co-cultured with *Tulasnella* sp. or *S. vermifera* were collected by tweezers and some of the intact seeds had their seed coat removed just before the SEM observation under a stereomicroscope (SZX16; Olympus).

## 5. Conclusions

Recruiting appropriate mycorrhizal fungi as symbiotic partners is indispensable for orchids to initiate the symbiotic seed germination. The process of the recruitment of symbiotic fungi at the symbiosis initiation stage remains unclear. In the present study, we compared seed germination between intact seeds and seed coat-stripped seeds using an experimental system for a symbiotic germination of a terrestrial orchid, *Bletilla striata* inoculated with symbiotic fungi *Tulasnella* sp. strain HR1-1 and *S. vermifera.* Our study demonstrated that seed coat-stripping treatment of the seeds resulted in a low germination rate and pathogen-like infection, even following inoculation with the symbiotic fungi *Tulasnella* sp. strain HR1-1 and *S. vermifera*. Moreover, when intact seeds were inoculated with the pathogenic fungi *R. solani* and *F. oxysporum*, hyphae were restricted to the suspensor end, as opposed to the seed coat-stripped seeds subjected to severe infestation. These results suggest that the *B. striata* seed coat plays a role in restricting ingress of fungal hyphae into the embryo cells, providing a barrier for uncontrolled plant tissue colonization. A similar analysis of other forms of orchid seeds would help improve understanding of the selection mechanism of the right partner among the soil microbiota and the potential adaptive significance of species diversity in orchids.

## Supplementary Materials

The following are available online at https://www.mdpi.com/2223-7747/8/8/280/s1, Figure S1: Seed germination of *Bletilla striata* in axenic culture, Figure S2: Seed germination of *Bletilla striata* inoculated with *Sebacina vermifera*, Figure S3: The criteria for infection of *Bletilla striata* seeds by pathogenic fungi *Rhizoctonia solani* or *Fusarium oxysporum*.
