# Supplementary material for: Bletilla striata (Orchidaceae) Seed Coat Restricts the Invasion of Fungal Hyphae at the Initial Stage of Fungal Colonization
Source: Plants (Basel). 2019 Aug 11;8(8):280. doi: 10.3390/plants8080280 (PMC6724134; doi:10.3390/plants8080280)
Supplement: Supplementary file 1 [file plants-08-00280-s001.pdf]

Supplementary materials

Plants

Bletilla striata (Orchidaceae) seed coat restricts the invasion of fungal hyphae at the initial stage of fungal colonization

Chihiro Miura, Miharu Saisho, Takahiro Yagame, Masahide Yamato, Hironori Kaminaka*

*Corresponding author:
Hironori Kaminaka
Faculty of Agriculture, Tottori University, Tottori, Japan
E-mail address: kaminaka@tottori-u.ac.jp

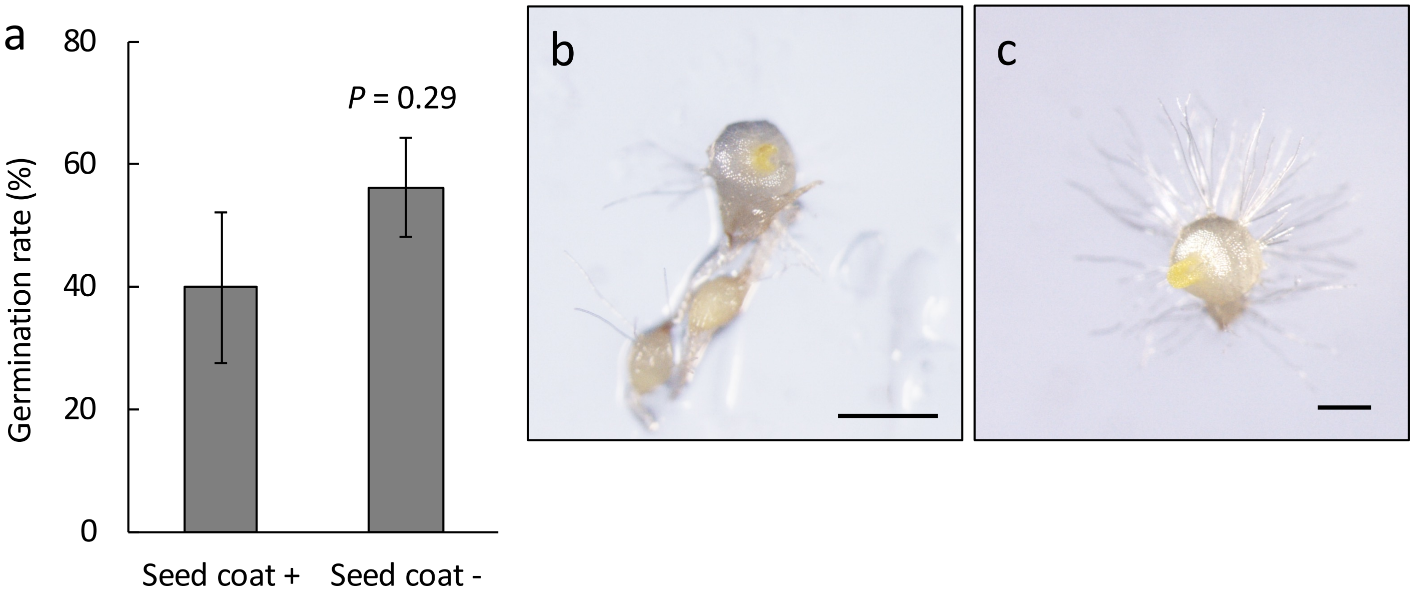

Figure S1. Seed germination of *Bletilla striata* in axenic culture. (**a**) Germination rate of *B. striata* seeds. Gray bars indicate the germination rate defined as the emergence of shoot apex at three weeks after sowing. Seed coat +, intact seeds; Seed coat -, seed coat-stripped seeds. Error bars represent standard errors of the means of seven independent experiments. *P* represents the *P* value for the difference between intact seeds and seed coat-stripped seeds determined by the Student's *t*-test. (**b, c**) The images show three-week-old protocorms of intact seed (**b**) and seed coat-stripped seed (**c**). Scale bars, 500 μm.

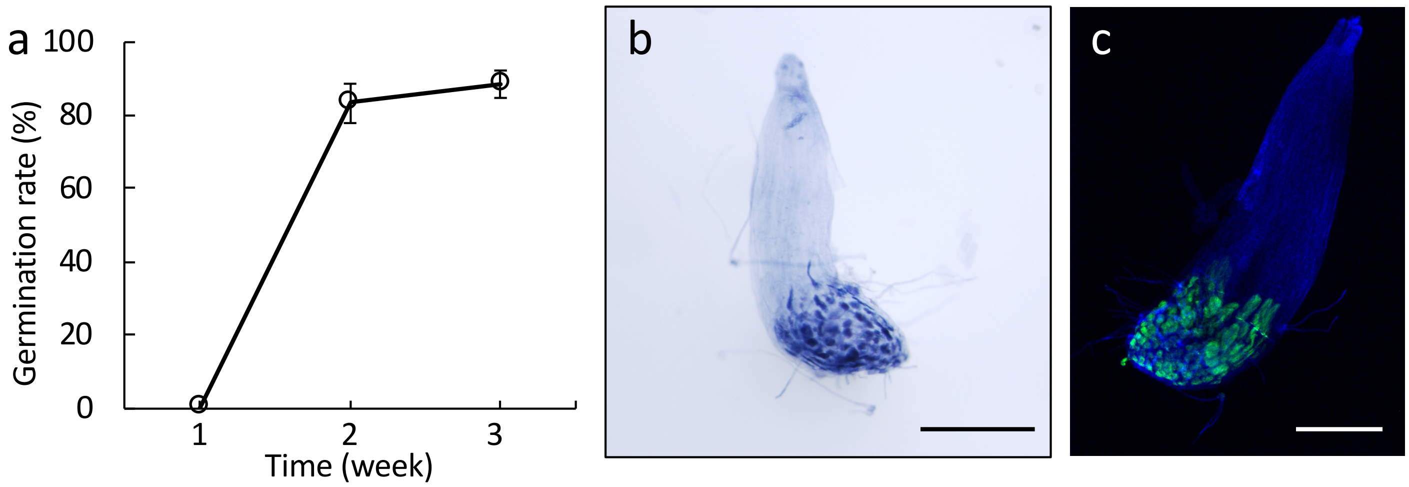

Figure S2. Seed germination of *Bletilla striata* inoculated with *Sebacina vermifera*. (**a**) Germination rate of *B. striata* seeds. The solid line indicates the germination rate defined as the emergence of shoot apex during the first three weeks after sowing. Error bars represent standard errors of the means of three independent experiments. (**b**) Colonization of a *B. striata* protocorm by *S. vermifera*. The image shows a two-week-old protocorm that was stained by ink solution (blue) to visualize fungal structures. The staining procedure was according to Yamamoto et al (2017). (**c**) A successfully germinated seed coat-stripped seed inoculated with *S. vermifera*. The image shows a three-weeks-old protocorm that was stained with calcofluor white (blue) and WGA-Alexa fluor-488 (green) to visualize the plant cell and fungal structures, respectively. Scale bars, 500 µm. Scale bars, 500 µm.

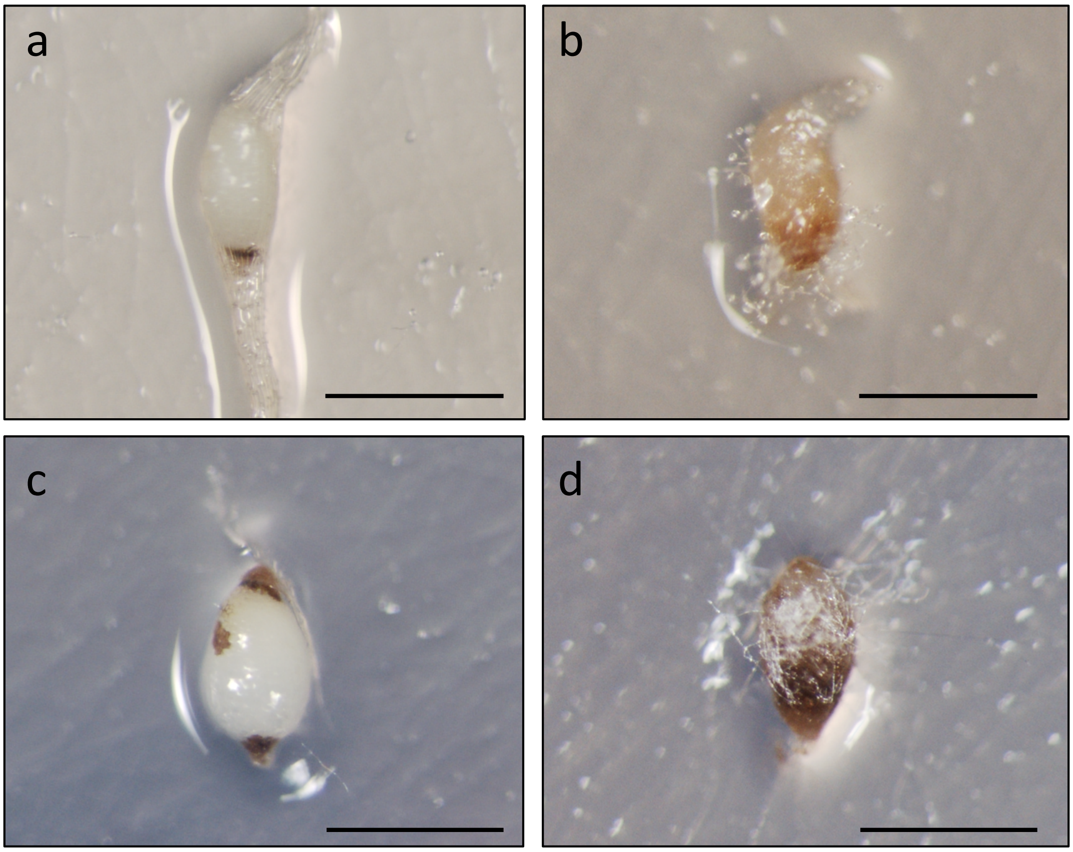

Figure S3. The criteria for infection of *Bletilla striata* seeds by pathogenic fungi *Rhizoctonia solani* (**a, b**) or *Fusarium oxysporum* (**c**, **d**). (**a**, **c**) Uninfected seeds. The seeds are creamy white in color with or without small brown dots. (**b**) *R. solani*-infected seed. The fungal hyphae cover the surface of the seed, and the seed color is creamy white or brown. (**d**) *F. oxysporum*-infected seed. The fungal hyphae cover the surface of the seed. The seed color changes from creamy white to dark purple or brown. Scale bars, 500 μm.
